# Effect of Polyethylene Glycol Loxenatide Treatment on Body Weight in Patients With Type 2 Diabetes With Different Body Mass Index: A Retrospective Real‐World Study

**DOI:** 10.1155/ije/7060233

**Published:** 2026-05-07

**Authors:** Peng Xu, Chen Dong, Xingzhi Xu, Kuanyong Zheng, Qinghua Liu, Yale Duan, Zhizhen Hu, Liya Bao, Benping Zhang

**Affiliations:** ^1^ The Second Clinical College, Tongji Medical College, Huazhong University of Science and Technology, Wuhan, Hubei, China, hust.edu.cn; ^2^ Department of Hepatobiliary Surgery and Liver Transplantation, Zhongshan Hospital, Fudan University, Shanghai, China, fudan.edu.cn; ^3^ Department of Pediatrics, Tongji Hospital, Tongji Medical College, Huazhong University of Science and Technology, Wuhan, Hubei, China, hust.edu.cn; ^4^ Department of Endocrinology, Xiantao First People’s Hospital, Xiantao, Hubei, China; ^5^ Department of Endocrinology, The Affiliated Hospital of Hubei University of Science and Technology, Xiantao, Hubei, China; ^6^ Department of Endocrinology, Tongji Hospital, Tongji Medical College, Huazhong University of Science and Technology, Wuhan, Hubei, China, hust.edu.cn; ^7^ Branch of National Clinical Research Center for Metabolic Diseases, Wuhan, Hubei, China; ^8^ Department of Medical Affairs, Jiangsu Hansoh Pharmaceutical Group Co., Ltd., Shanghai, China; ^9^ Hubei Clinical Medical Research Center for Endocrinology and Metabolic Diseases, Wuhan, Hubei, China

**Keywords:** body mass index, body weight, glucagon-like peptide-1 receptor agonists, PEG-Loxe, polyethylene glycol loxenatide, Type 2 diabetes mellitus

## Abstract

**Introduction:**

Real‐world data on the effect of polyethylene glycol loxenatide (PEG‐Loxe) in patients with Type 2 diabetes mellitus (T2DM) with normal body mass index (BMI) are lacking. Therefore, this study aims to observe the effect of PEG‐Loxe on the weight of patients with T2DM with different BMIs.

**Methods:**

This single‐center, retrospective real‐world study included patients with T2DM, aged ≥ 18 years, and receiving PEG‐Loxe/sodium‐glucose cotransporter 2 inhibitor (SGLT2i). The primary endpoint was change in body weight after 24 weeks of treatment. Secondary endpoints included proportion of patients with body weight reduction < 5.0% and < 10%, changes in BMI, and glycosylated hemoglobin (HbA1c).

**Results:**

Overall, 487 patients treated with SGLT2i or PEG‐Loxe were identified; after propensity score matching, 68 patients were included in each group. After 24 weeks, the mean change in body weight and BMI was −1.2 (−2.3, −0.1) and −0.4 (−0.8, −0.1) in the PEG‐Loxe group and −2.4 (−3.5, −1.3) and ‐0.7 (−1.1, −0.4) in the SGLT2i group. A ≥ 5% and ≥ 10% weight loss was noted by 32.4% and 8.8% of patients in the PEG‐Loxe group, with a mean change in HbA1c (%) of −1.48 (−1.98, −0.99). Weight loss (≥ 5%) in obese patients (BMI > 28 kg/m^2^) was greater (PEG‐Loxe: 53.3%, SGLT2i: 57.1%) compared to patients with 24 ≤ BMI < 28 kg/m^2^ (PEG‐Loxe: 25.7%, SGLT2i: 41.4%) and BMI < 24 kg/m^2^ (PEG‐Loxe: 27.8%, SGLT2i: 28%).

**Conclusion:**

PEG‐Loxe resulted in weight loss and glycemic control in patients with T2DM. The weight loss was negatively correlated with baseline BMI without excessive weight loss in normal BMI, supporting PEG‐Loxe’s use in this group.

**Trial Registration:** Chinese Registry of Clinical Trials: ChiCTR2400087532

## 1. Introduction

Type 2 diabetes mellitus (T2DM) is commonly associated with risk factors such as overweight and obesity [1]. According to the International Diabetes Federation (IDF), as of 2021, China has the largest number of people with T2DM globally (141 million) with a prevalence of 13% [2–4]. In addition, more than half of patients with T2DM in China are overweight or obese, placing a considerable burden on its healthcare system [5].

Among the drugs available to treat T2DM, glucagon‐like peptide‐1 receptor agonists (GLP‐1 RAs) have garnered attention over the past few years owing to their effective glycemic control as well as weight loss [6]. In addition to augmenting insulin secretion and inhibiting glucagon production from pancreatic alpha cells, GLP‐1 RAs lower blood pressure and total cholesterol (TC) and exert positive effects on coronary heart disease and kidney function in patients with T2DM [7, 8]. To date, the US Food and Drug Administration has recognized three classes of antidiabetic drugs—sodium‐glucose cotransporter two inhibitors (SGLT2i), dipeptidyl peptidase four inhibitors (DPP‐4i), and GLP‐1 RAs—for meeting their established cardiovascular safety endpoints with noninferiority to placebo [9]. Numerous cardiovascular outcome trials have demonstrated that GLP‐1 RAs (such as semaglutide, dulaglutide, efpeglenatide, and liraglutide) and SGLT2i reduce the incidence of major adverse cardiovascular events in patients with T2DM [10–13].

In contrast to the patients in Western countries, a large proportion of patients with T2DM in China are of normal weight (body mass index [BMI], 18.5–24.9 kg/m^2^) [14]. Due to the difference in the baseline BMI of these two populations, studies have shown that GLP‐1 RAs exert a hypoglycemic effect more effectively in Asians than in non‐Asians [15].

Of note, it has been found that BMI and mortality are inversely associated with a lower risk of death in overweight (BMI 25.0–29.9 kg/m^2^) and obese (BMI ≥ 30 kg/m^2^) patients with T2DM, compared with normal weight patients [16]. This raises concerns among clinicians regarding the possibility of excessive weight loss in normal weight patients with T2DM receiving GLP‐1 RA.

Polyethylene glycol loxenatide (PEG‐Loxe) is a once‐weekly GLP‐1 RA formulation derived from amino acid and polyethylene glycol (PEG) modifications of exendin‐4 [17]. PEG‐Loxe injection is the first self‐developed long‐term GLP‐1 RA in China [18] and was approved by the National Medical Products Administration (NMPA) for release in the Chinese market in 2019 [19].

In addition to its ability to effectively control blood glucose, PEG‐Loxe is also able to reduce weight in overweight and obese patients with T2DM [20]. However, no studies have reported the effect of PEG‐Loxe on weight loss in patients with normal BMI in real‐world conditions. This study aimed to assess the effect of PEG‐Loxe treatment on the body weight of patients with T2DM having different BMIs.

## 2. Methods

### 2.1. Study Design

This retrospective, real‐world study collected data from the electronic medical record system of Tongji Hospital, affiliated with Tongji Medical College of Huazhong University of Science and Technology, from 1 January 2019 to 31 June 2024. An informed consent was waived for the study as reviewed and approved by the Ethics Committee of Tongji Hospital affiliated to Tongji Medical College of Huazhong University of Science and Technology). The study was conducted in accordance with the Declaration of Helsinki and is registered in ClinicalTrials.gov.

### 2.2. Patient Population and Data Collection

The study included patients with T2DM aged ≥ 18 years who had received PEG‐Loxe or SGLT2i treatment for at least 24 weeks. Patients with type 1 diabetes or those who had received PEG‐Loxe in combination with SGLT2i were excluded. Baseline data such as age, sex, weight, BMI, glycosylated hemoglobin (HbA1c), fasting blood glucose (FPG), blood pressure (systolic blood pressure [SBP] and diastolic blood pressure [DBP]), blood lipids (TC, triglyceride [TG], high‐density lipoprotein cholesterol [HDL‐C], low‐density lipoprotein cholesterol [LDL‐C]), and use of hypoglycemic drugs were collected at the start of treatment (PEG‐Loxe or SGLT2i). Patients treated with SGLT2i served as control and were matched with the PEG‐Loxe group by propensity score matching (PSM) in a 1:1 ratio.

### 2.3. Study Endpoints

The primary endpoint of the study was the change in body weight from baseline after treatment with PEG‐Loxe and SGLT2i for 24 weeks. Secondary endpoints included the proportion of patients with weight loss ≥ 5% and ≥ 10%; changes in BMI, HbA1c, FPG, blood pressure, and blood lipids from baseline after 24 weeks of treatment.

### 2.4. Statistical Methods

The normal distribution of continuous variables was tested using the Kolmogorov–Smirnov test. An independent samples *t*‐test or Mann–Whitney *U* test or chi‐square test was used to identify baseline characteristics. PSM was conducted for logistic regression to adjust for potential confounding factors (1:1 ratio using a caliper = 0.4 algorithm; covariates: age, sex, baseline BMI, and HbA1c) ensuring comparability between the groups. The change in body weight from baseline to Week 24 was analyzed using the analysis of covariance (ANCOVA) model (age, sex, and baseline BMI were the covariates). A similar ANCOVA model was used to analyze the proportion of patients with BMI, HbA1c, FPG, blood pressure, and blood lipids. For analysis of categorical variables, the chi‐square test was used. The statistical significance of the results was defined as *p* < 0.05. SPSS 27.0 software was used for statistical analysis.

## 3. Results

### 3.1. Baseline Characteristics

**FIGURE 1:**
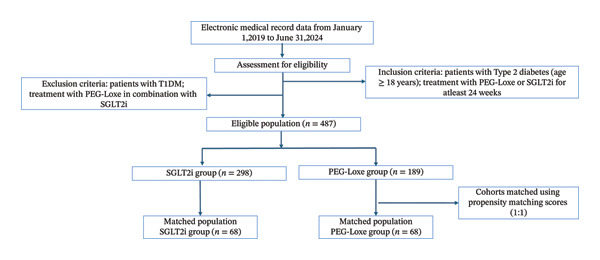
Patient disposition. Abbreviations: HbA1c, glycosylated hemoglobin; PEG‐Loxe, polyethylene glycol loxenatide; SGLT2i, sodium‐glucose cotransporter 2 inhibitors.

A total of 487 patients with T2DM treated with SGLT2i or PEG‐Loxe were identified, and 68 of these patients were classified into the PEG‐Loxe group after PSM. An additional 68 SGLT2i‐treated patients were matched as controls (Figure 1).

In the PEG‐Loxe group, 22.1% were female patients with a mean (standard deviation [SD]) age of 54.7 (10.4) years, a mean (SD) body weight of 73.8 (12.0) kg, and a mean BMI of 26.1 (3.3) kg/m^2^. The mean baseline HbA1c and median (IQR) FPG levels were 8.95% (SD: 2.20%) and 10.34 mmol/L (range: 8.43, 12.80), respectively. The mean (SD) SBP and DBP were 131.1 (14.0) and 83.5 (11.4) mm Hg, respectively, with the TG level of 2.07 (range: 1.32, 3.46) mmol/L. At baseline, 60.3% of patients received treatment with biguanides, 14.7% with sulfonylureas/biglinides, 39.7% with thiazolidinediones, and 83.8% with insulin (Table 1).

**TABLE 1:** Baseline characteristics of patients before and after propensity score matching.

	**Before propensity score matching**			**After propensity score matching**		
	**PEG-Loxe**	**SGLT2i**	**p** **value**	**PEG-Loxe**	**SGLT2i**	**p** **value**

*n*	189	298		68	68	
Women, *n* (%)	39 (20.1)	112 (37.7)	< 0.001	15 (22.1)	16 (23.5)	0.838
Age, years	50.8 (10.5)	59.2 (10.7)	< 0.001	54.7 (10.4)	53.8 (10.4)	0.603
Body weight (kg)	75.5 (12.2)	66.7 (10.8)	< 0.001	73.8 (12.0)	72.3 (11.8)	0.482
BMI (kg/m^2)^	26.5 (3.4)	24.2 (3.1)	< 0.001	26.1 (3.3)	25.3 (3.2)	0.150
HbA1c (%)	9.50 (2.31)	8.53 (2.24)	< 0.001	8.95 (2.20)	8.95 (2.32)	0.997
FPG (mmol/L)	11.30 (8.87, 14.76)	11.18 (8.19, 16.51)	0.965	10.34 (8.43, 12.80)	11.38 (8.36, 17.15)	0.082
SBP (mm Hg)	130.1 (15.0)	131.2 (21.9)	0.528	131.1 (14.0)	129.7 (19.3)	0.637
DBP (mmHg)	84.0 (11.1)	80.1 (12.4)	< 0.001	83.5 (11.4)	82.9 (13.7)	0.749
TC (mmol/L)	4.56 (1.13)	4.40 (1.42)	0.213	4.39 (1.02)	4.55 (1.40)	0.450
TG (mmol/L)	2.44 (1.66, 3.87)	1.84 (1.29, 3.00)	< 0.001	2.07 (1.32, 3.46)	2.27 (1.37, 3.31)	0.692
HDL‐C (mmol/L)	0.97 (0.24)	1.06 (0.30)	< 0.001	1.01 (0.24)	0.96 (0.22)	0.269
LDL‐C (mmol/L)	2.54 (2.08, 3.29)	2.44 (1.72, 3.32)	0.168	2.45 (1.79, 3.27)	2.45 (1.83, 3.39)	0.853
Antidiabetic drugs, *n* (%)						
Biguanides	136 (72.0)	143 (48.0)	< 0.001	41 (60.3)	46 (67.6)	0.372
SU/glinides	32 (16.9)	42 (14.1)	0.395	10 (14.7)	11 (16.2)	0.812
AGI	56 (29.6)	137 (46.0)	< 0.001	24 (35.3)	34 (50.0)	0.083
TZD	104 (55.0)	52 (17.4)	< 0.001	27 (39.7)	29 (42.6)	0.727
Insulin	164 (86.8)	248 (83.2)	0.290	57 (83.8)	55 (80.9)	0.653

*Note:* Data are expressed as mean (SD) or median (interquartile range), unless otherwise indicated. FPG, fasting blood glucose; HbA1c, glycosylated hemoglobin.

Abbreviations: BMI, body mass index; DBP, diastolic blood pressure; HDL‐C, high‐density lipoprotein cholesterol; LDL‐C, low‐density lipoprotein cholesterol; SBP, systolic blood pressure; SU, sulfonylureas; TC, total cholesterol; TG, triglyceride; TZD, thiazolidinedione.

### 3.2. Efficacy Outcomes

After 24 weeks of treatment with PEG‐Loxe and SGLT2i, a mean reduction of −1.2 (95% CI: −2.3, −0.1) and −2.4 (95% CI: −3.5, −1.3) was observed in the body weight of the patients, respectively. The intergroup difference was 1.2 kg (95% CI: −0.4, 2.8; *p* = 0.141).

A total of 32.4% of patients in the PEG‐Loxe group and 39.7% in the SGLT2i group had a weight loss of ≥ 5%. A weight loss of ≥ 10% was observed in 8.8% and 10.3% patients in the PEG‐Loxe group and the SGLT2i group, respectively.

The BMI changes from baseline to 24 weeks were −0.4 (95% CI: −0.8, −0.1) and −0.7 (95% CI: −1.1, −0.4) in patients treated with PEG‐Loxe and SGLT2i, respectively, with no statistically significant difference between the two groups (*p* = 0.275).

The HbA1c levels decreased by 1.48% and 1.42% in the PEG‐Loxe and SGLT2i groups, respectively (*p* = 0.64). Similarly, FPG decreased by 3.83 and 2.01 mmol/L, respectively, in patients treated with PEG‐Loxe and SGLT2i, with no significant intergroup differences (*p* = 0.59).

In terms of blood lipids and blood pressure, TG levels decreased by 0.05 mmol/L (95% CI: −0.50, 0.73) and −0.05 mmol/L (95% CI: −1.63, 0.79) in the PEG‐Loxe and SGLT2i groups, respectively. The intergroup difference was 0.10 mmol/L (95% CI: −0.35, 0.82) and was nonsignificant (*p* = 0.481). Changes in SBP, DBP, TC, HDL‐C, and LDL‐C were not significant between the two groups (all *p* > 0.05) (Table 2).

**TABLE 2:** Comparison of trial outcome measures between PEG‐Loxe and SGLT2i after 6 months of treatment.

	**PEG-Loxe (*n* = 68)** **Mean (95% CI)**	**SGLT2i (*n* = 68)** **Mean (95% CI)**	**Between-group** **Difference (95% CI)**	**p** **value**

Change in body weight (kg)	−1.2 (−2.3, −0.1)	−2.4 (−3.5, −1.3)	1.2 (−0.4, 2.8)	0.141
Change in BMI (kg/m^2)^	−0.4 (−0.8, −0.1)	−0.7 (−1.1, −0.4)	0.3 (−0.2, 0.9)	0.275
Patients with ≥ 5% weight loss, *n* (%)	22 (32.4)	27 (39.7)	—	0.372
Patients with ≥ 10% weight loss, *n* (%)	6 (8.8)	7 (10.3)	—	0.771
Change in HbA1c (%)	−1.48 (−1.98, −0.99)	−1.42 (−1.91, −0.93)	−0.06 (−0.76, 0.64)	0.856
Change in FPG (mmol/L)	−3.83 (−5.83, −1.50)	−2.01 (−6.44, 0.44)	−1.82 (−3.14, 0.59)	0.170
Change in SBP (mmHg)	−4.0 (−8.1, 0.1)	−5.6 (−9.3, −1.9)	1.6 (−3.9, 7.1)	0.564
Change in DBP (mmHg)	−1.1 (−3.9, 1.6)	−4.5 (−6.9, −2.0)	3.4 (−0.3, 7.0)	0.073
Change in TC (mmol/L)	−0.16 (−0.45, 0.13)	−0.37 (−0.62, −0.12)	0.21 (−0.18, 0.60)	0.285
Change in TG (mmol/L)	0.05 (−0.50, 0.73)	−0.05 (−1.63, 0.79)	0.10 (−0.35, 0.82)	0.481
Change in HDL‐C (mmol/L)	0.11 (0.04, 0.17)	0.10 (0.05, 0.16)	0.00 (−0.08, 0.09)	0.923
Change in LDL‐C (mmol/L)	0.07 (−0.80, 0.63)	−0.05 (−0.63, 0.33)	0.12 (−0.37, 0.47)	0.734

*Note*: Data are expressed as mean (SD) or median (interquartile range), unless otherwise indicated. FPG, TG, and LDL‐C were analyzed by Mann–Whitney *U* test. HbA1c, glycosylated hemoglobin; FPG, fasting blood glucose.

Abbreviations: BMI, body mass index; CI, confidence interval; DBP, diastolic blood pressure; HDL‐C, high‐density lipoprotein cholesterol; LDL‐C, low‐density lipoprotein cholesterol; SBP, systolic blood pressure; TC, total cholesterol; TG, triglyceride.

On analyzing the effects of PEG‐Loxe and SGLT2i treatment on the body weight of patients with different BMIs, it was observed that the weight loss was directly proportional to the baseline BMI. In patients treated with PEG‐Loxe, a minimal decrease of −0.4 kg (95% CI: −2.1, 2.9) was observed in the body weight of the patients with BMI < 24 kg/m^2^, whereas a maximum decrease of −4.0 kg (95% CI: −6.7, −1.3) in body weight was observed in obese patients with BMI > 28 kg/m^2^. A similar trend was observed in patients treated with SGLT2i, with a decrease in body weight of −1.0 (95% CI: −3.1, 1.2) and −4.2 kg (95% CI: −7.0, −1.4) in patients with BMI < 24 and BMI > 28 kg/m^2^, respectively (Table 3). Moreover, a negative correlation was observed between change in body weight and baseline BMI (*r* = −0.28, *p* = 0.019) (Figure 2).

**TABLE 3:** Change in body weight from baseline to Week 24, stratified by baseline BMI level.

	**PEG-Loxe** **Mean (95% CI)**	**SGLT2i** **Mean (95% CI)**	**Between-group** **Difference (95% CI)**	**p** **value**

BMI < 24 kg/m^2^	−0.4 (−2.1, 2.9) N = 18	−1.0 (−3.1, 1.2) N = 25	1.4 (−2.0, 4.8)	0.415

24 ≤ BMI < 28 kg/m^2^	−1.2 (−2.5, 0.2) N = 35	−2.3 (−3.8, −0.8) N = 29	1.1 (−1.0, 3.1)	0.291

BMI > 28 kg/m^2^	−4.0 (−6.7, −1.3) N = 15	−4.2 (−7.0, −1.4) N = 14	0.2 (−3.8, 4.1)	0.929

Abbreviation: BMI, body mass index.

FIGURE 2Correlation between baseline BMI and change in body weight. (A) PEG‐Loxe group. (B) SGLT2i group. Abbreviation: BMI, body mass index.

**Figure:**
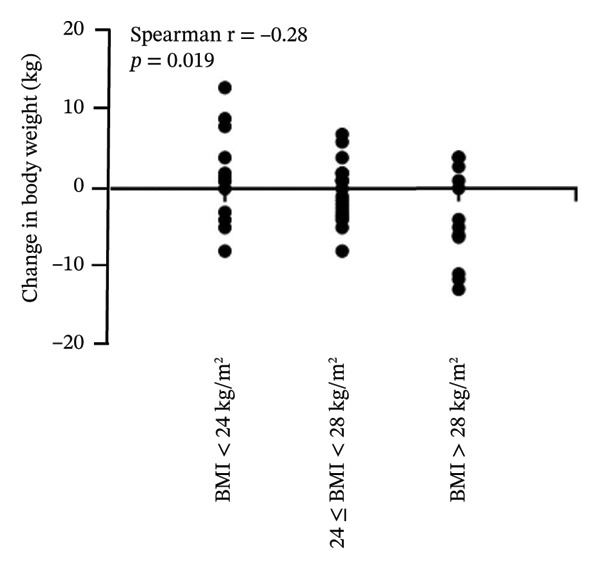
(A)

**Figure:**
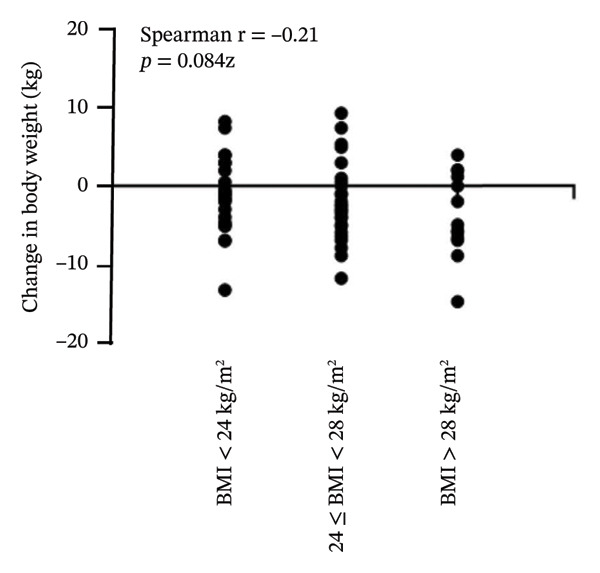
(B)

Upon analyzing the difference in weight loss (≥ 5% and ≥ 10%) in patients with different BMIs, it was observed that the proportion of patients achieving ≥ 5% weight loss increased with increased BMI. A total of 27.8% and 28% of patients with a BMI < 24 kg/m^2^ experienced ≥ 5% weight loss after receiving treatment with PEG‐Loxe and SGLT2i, respectively. A relatively higher percentage of patients with BMI > 28 kg/m^2^ experienced ≥ 5% weight loss after treatment with PEG‐Loxe and SGLT2i (53.3% vs 57.1%). When compared with 41.4% of normal weight patients (24 ≤ BMI < 28 kg/m^2^) treated with SGLT2i, only 25.7% of patients treated with PEG‐Loxe experienced a weight loss of ≥ 5%. Of note, the intergroup differences were not significant for all different BMI categories (all *p* > 0.05) (Figure 3).

FIGURE 3Proportion of patients achieving thresholds of weight loss. (A) Patients with ≥ 5% weight loss. (B) Patients with ≥ 10% weight loss. Abbreviations: BMI, body mass index; PEG‐Loxe, polyethylene glycol loxenatide; SGLT2i, sodium‐glucose cotransporter two inhibitors.

**Figure:**
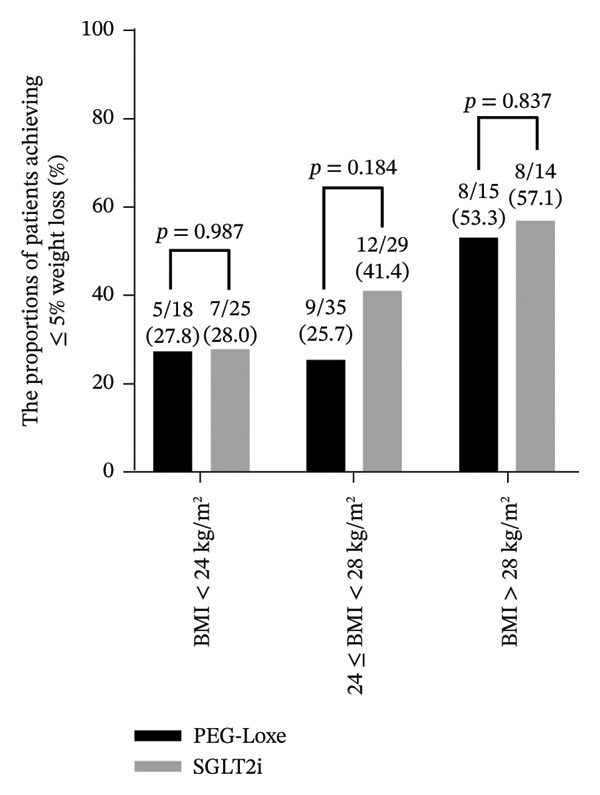
(A)

**Figure:**
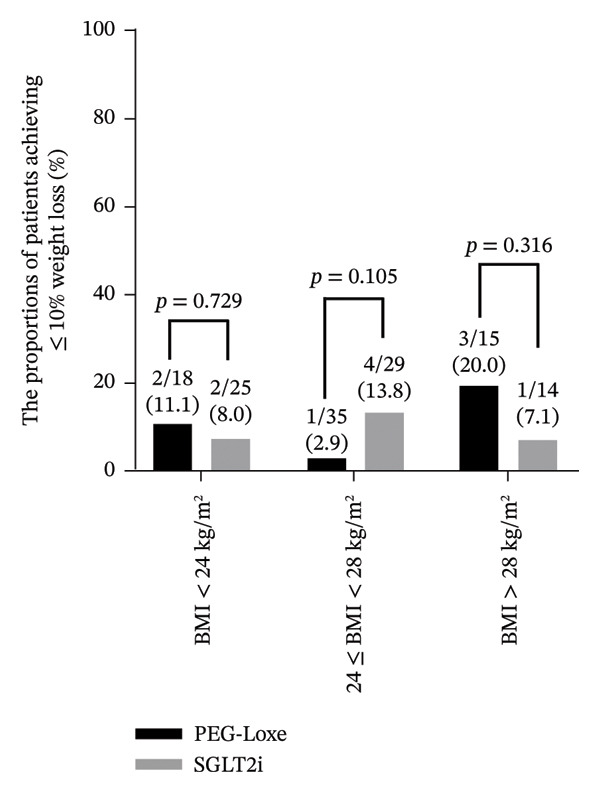
(B)

*A* ≥ 10% weight loss was observed in 11.1% and 8% of patients with BMI < 24 kg/m^2^ and 20% and 7.1% of patients with BMI > 28 kg/m^2^ after receiving treatment with PEG‐Loxe and SGLT2i, respectively. Notably, a higher proportion of patients with normal body weight experienced a weight loss of ≥ 10% after treatment with SGLT2i (13.8%) compared with PEG‐Loxe (2.9%). However, the intergroup differences among patients in the three BMI categories were not significant (all *p* > 0.05) (Figure 3).

## 4. Discussion

This retrospective cohort study demonstrated the effects of PEG‐Loxe in comparison with SGLT2i on body weight, blood glucose, blood pressure, and blood lipids in patients with T2DM having different BMIs. After 24 weeks of treatment, patients treated with PEG‐Loxe showed a mean reduction of −1.2 kg in body weight. Moreover, fewer patients in the PEG‐Loxe group (32.4% and 8.8%) had ≥ 5% and ≥ 10% weight loss compared with the SGLT2i group (39.7% and 10.3%). There were no statistically significant intergroup differences in the BMI, HbA1c, blood pressure, and blood lipids. In addition, weight loss was directly proportional to the baseline BMI, with obese patients having the maximum decrease in body weight.

By acting on central mechanisms, GLP‐1 RAs reduce appetite, increase satiety, lower caloric intake, and contribute to weight reduction [21]. On an average, GLP‐1 RA results in a weight reduction of 2 to 5 kg in patients with T2DM [22]. In a phase 1 trial, administration of 0.3 mg PEG‐Loxe resulted in a weight reduction by 1.9 kg in nonobese patients with T2DM [23]. After 24 weeks of treatment with PEG‐Loxe and SGLT2i, a reduction of 1.2 and 2.4 kg in the body weight of the patients was observed in our study, with a statistically nonsignificant intergroup variation. This suggests that PEG‐Loxe is effective as a weight loss agent in patients who are overweight.

A phase 3a study reported that 24‐week PEG‐Loxe monotherapy in patients with T2DM showed promising efficacy, as observed by reductions of 1.02% to 1.34% in HbA1c levels [24]. Furthermore, in a phase 3b study, PEG‐Loxe at doses of 100 and 200 μg in combination with metformin has shown to reduce HbA1c levels by −1.16% to −1.14% [25]. Treatment with 100 and 200 μg PEG‐Loxe for 12 weeks decreased HbA1c levels by −1.02% and −1.36% from baseline, respectively [26]. These results are comparable with our study, in which HbA1c levels decreased by 1.48% in patients treated with PEG‐Loxe.

Our study also reported a total of 32.4% patients in the PEG‐Loxe group and 39.7% in the SGLT2i group with a weight loss of ≥ 5%. Furthermore, a weight loss of ≥ 10% was observed in 8.8% and 10.3% patients in the PEG‐Loxe group and the SGLT2i group, respectively. The results of our study are comparatively lower than those of Cai et al., where treatment with PEG‐Loxe for 16 weeks resulted in 61.5% and 26.9% of patients with weight loss of ≥ 5% and ≥ 10% [5].

In our study, 24 weeks of PEG‐Loxe and SGLT2i treatment resulted in a change in baseline BMI by −0.4 and −0.7 kg/m^2^, respectively (not significant); FPG decreased by 3.83 and 2.01 mmol/L, respectively. Similarly, a 16 week treatment with PEG‐Loxe resulted in a change of −2.55 kg/m^2^ in the baseline BMI with no significant reduction in the mean FPG [5]. A 3–6‐month treatment with GLP‐1 RA reduced FPG by 2.3 mmol/L [27]. The current results also corroborate the findings that treatment with PEG‐Loxe can reduce TC, TG, LDL‐C levels, SBP, and DBP [5].

In our study, weight loss was negatively correlated with the baseline BMI. In PEG‐Loxe‐ and SGLT2i‐treated patients, a minimal decrease in body weight was observed in patients with BMI < 24 kg/m^2^, whereas the maximum decrease was observed in obese patients. In a randomized‐controlled trial, 16 weeks of PEG‐Loxe treatment resulted in an average weight loss of 7.52 kg in patients with T2DM who were overweight or obese [5]. However, there is no real‐world evidence on the effect of PEG‐Loxe injection on the body weight of patients with T2DM having normal BMIs.

In general, BMI has been reported to have a “J” shaped association with overall mortality [28], warranting appropriate weight management in patients with T2DM. Upon analyzing the difference in weight loss (≥ 5% and ≥ 10%) in patients with different BMIs, it was observed that the proportion of patients achieving a ≥ 5% weight loss increased with increased BMI. When compared with 41.4% of normal weight patients treated with SGLT2i, only 25.7% of patients treated with PEG‐Loxe experienced ≥ 5% weight loss. A higher proportion of patients with normal body weight experienced ≥ 10% weight loss after treatment with SGLT2i (13.8%) compared with patients treated with PEG‐Loxe (2.9%). This weaker weight loss effect of PEG‐Loxe is appropriate, as it supports safe weight management while prioritizing overall health and effective diabetes control. Patients with normal BMI typically have less excess weight to lose, and any weight loss experienced by them might not be clinically significant or beneficial. For these patients, the primary focus of treatment should be on effective diabetes management rather than achieving substantial weight reduction [29]. Therefore, a weaker weight loss effect is not only appropriate but may also be a safer option, ensuring that these patients remain within a healthy weight range while effectively managing their blood glucose levels.

The study, however, has some limitations. The limited sample size (especially in BMI‐stratified subgroups) renders secondary endpoint and subgroup analyses exploratory, necessitating validation in larger cohorts. Furthermore, despite the presence of a control group, the retrospective nature of the study may have led to the risk of bias in evaluation. Evidence from long‐term PEG‐Loxe treatment studies is therefore warranted.

## 5. Conclusion

Treatment of T2DM patients with PEG‐Loxe has the effect of weight loss and glycemic control, and in patients with different BMIs, weight loss is negatively correlated with baseline BMI. As a result, the treatment of patients with T2DM and normal BMI with PEG‐Loxe does not increase the risk of excessive weight loss. This study provides a therapeutic basis for the application of PEG‐Loxe in T2DM patients with normal BMI.

## Author Contributions

Peng Xu, Chen Dong, and Benping Zhang contributed to methodology, data curation, formal analysis, and original draft preparation. Xingzhi Xu and Qinghua Liu contributed to designing the methodology and data analysis. Yale Duan, Zhizhen Hu, and Liya Bao contributed to critical review of the manuscript. Benping Zhang contributed to data interpretation, project administration, funding acquisition, manuscript review, conceptualization, and supervision.

## Funding

This work was supported by the National Natural Science Foundation of China (No. 81700207).

## Ethics Statement

An informed consent was waived off for the study as reviewed and approved by the Ethics Committee of Tongji Hospital affiliated to Tongji Medical College of Huazhong University of Science and Technology (No. TJ‐IRB202406061). The study was conducted in accordance with the Declaration of Helsinki and is registered in ClinicalTrials.gov (ChiCTR2400087532).

## Conflicts of Interest

Yale Duan, Zhizhen Hu, and Liya Bao are employed by Jiangsu Hansoh Pharmaceutical Group Co., Ltd. The other authors declare no conflicts of interest.

## Data Availability

The data that support the findings of this study are available from the corresponding author upon reasonable request.
